# 50% body weight loading reduces stature increases and lumbar disc expansion from 4 h hyper‐buoyancy floatation versus 15 min sitting upright

**DOI:** 10.1113/EP091745

**Published:** 2024-12-04

**Authors:** David Marcos‐Lorenzo, Christina Lysandrou, Laura Sudres, Alfonso Gil‐Martinez, Jaap Swanenburg, James Edward Clark, David Andrew Green

**Affiliations:** ^1^ School of Medicine of Autonomous University of Madrid Madrid Spain; ^2^ Centre of Human and Applied Physiological Sciences King's College London London UK; ^3^ Department of Physiotherapy Centro de Estudios Superiores La Salle Madrid Spain; ^4^ CranioSPain Research Group Centro Superior de Estudios Universitarios La Salle, Universidad Autónoma de Madrid Madrid Spain; ^5^ Unit of Physiotherapy, Hospital Universitario La Paz, Carlos III Institute for Health Research Madrid Spain; ^6^ Integrative Spinal Research ISR, Department of Chiropractic Medicine Balgrist University Hospital Zurich Switzerland; ^7^ Faculty of Medicine, Institute of Anatomy University of Zurich Zurich Switzerland; ^8^ Innovation Cluster Space and Aviation (UZH Space Hub), Air Force Center University of Zurich Dübendorf Switzerland; ^9^ KBRwyle GmbH Cologne Germany; ^10^ Institute for Risk and Disaster Reduction University College London (UCL) London UK; ^11^ Space Medicine Team, European Astronaut Centre European Space Agency Cologne Germany

**Keywords:** back pain, intervertebral disc, reloading, unloading

## Abstract

Microgravity is associated with stature increases, back pain and post‐flight intervertebral disc (IVD) herniation. This study aims to determine whether 30 s seated 50% body weight (BW) axial loading is comparable to 15 min sitting upright in 1 *g* upon changes in stature, anterior lumbar IVD height (via ultrasound), passive vertebral stiffness (VS), and back pain induced by 4 h hyper‐buoyancy floatation (HBF) unloading. Sixteen (seven male) healthy volunteers had stature, lumbar IVD height (L2–S1), passive VS (C1–L5) and back pain assessed before and following 4 h HBF, and immediately after participants performed a 30 s seated squat with 50% of their BW or 15 min sitting upright. Four hours of HBF unloading induced significant increments in stature (+1.6 ± 0.5 cm; *P *< 0.001), IVD height (L2–L3: *P* = 0.002; L3–L4: *P *< 0.001; L4–L5: *P* = 0.013; L5–S1: *P *< 0.001) and back pain (2.90 ± 1.26; *P *< 0.001) with no differences between 1 and 1.5 BW. Stature, IVD height increments and back pain were similarly attenuated in both reloading groups. Passive VS was unchanged by 4 h HBF or reloading. HBF‐induced back pain positively correlated with stature (*P* = 0.01) and lumbar IVD height changes (L2–L3: *P* = 0.03; L3–L4: *P* = 0.01; L5–S1: *P* = 0.02). Four hours of HBF increased stature, lumbar IVD height and induced moderate back pain that were similarly (albeit not entirely) ameliorated by both 15 min upright sitting and 30 s of 50% BW axial loading, with no changes in passive VS observed. IVD geometric changes appear key to space adaptation back pain and stature increments that can be rapidly modulated by brief periods of axial loading.

## Abstract (highlights)

- **What is the central question of this study?**
  Microgravity is associated with stature increases, back pain and an increased risk of post‐flight intervertebral disc herniation: is 30 s of seated 50% body weight axial loading comparable to 15 min of sitting upright in 1 *g* upon changes in stature, anterior lumbar intervertebral disc height (via ultrasound), passive vertebral stiffness, and back pain induced by 4 h hyper‐buoyancy floatation unloading?
- **What is the main finding and its importance?**
  30s of 50% body weight axial loading and 15 min sitting upright similarly ameliorated increased stature, anterior lumbar IVD and back pain, with no changes in passive vertebral stiffness observed. IVD geometric changes may underpin space adaptacion back pain and stature increments that can be modulated by brief periods of axial loading.

## 1 INTRODUCTION

Exposure to microgravity is associated with anthropometric changes (Thornton et al., 1977) including stature increases of up to 7 cm (Brown, 1977), and in the first few hours moderate‐to‐severe back pain (Pool‐Goudzwaard et al., 2015), reported by more than half of astronauts (Kerstman et al., 2012; Wing et al., 1991). In addition, several astronauts have experienced difficulties fitting into extra‐vehicular activity (EVA) suits, and their bespoke Soyuz ‘Kazbek’ seat pan (Nicogossian et al., 2016). However, the pathophysiology of ‘space adaptation back pain’ (Penchev et al., 2021) is largely unknown (Green & Scott, 2018), although spaceflight is associated with intervertebral disc (IVD) swelling (Sayson & Hargens, 2008), spinal curvature flattening (Andreoni et al., 2000) and reduced para‐spinal muscle tone (McNamara et al., 2019).

Furthermore, following long duration (>6 month) International Space Station (ISS) missions, trunk muscle atrophy (Burkhart et al., 2019; Chang et al., 2016) and dysfunction (Hides et al., 2021) associated with impaired spinal kinematics, spinal endplate irregularities, and segmental changes in spinal stiffness have been noted (Bailey et al., 2022). Such changes may contribute to an apparent increased risk of post‐flight IVD herniation (Johnston et al., 2010), an event that is debilitating on Earth, but could be critical when landing on the Moon. In fact, a spectrum of lumbar IVD pathologies have been observed with magnetic resonance imaging (MRI) following long duration spaceflight (Bailey et al., 2022; Chang et al., 2016) including disc desiccation and osteophytes (Garcia et al., 2018).

However, despite the potential significance, few studies evaluating the spinal column have been performed in‐flight due to the operational challenges of spinal imaging (Green & Scott, 2018). Vertebral stiffness (VS), defined as the vertebral column's resistance to deformation, has been proposed as a ‘global’ measure of vertebral function and has been performed in microgravity during parabolic flight (Glaus et al., 2021), albeit not in space. In fact, changes in body position (Häusler et al., 2020) have been shown to modulate vertebral (cervical, thoracic and lumbar) stiffness such that the term ‘active’ – reflecting activation of trunk musculature – is used when weight‐bearing, and ‘passive’ when not weight‐bearing, for example, when lying prone (Stokes & Gardner‐Morse, 2003).

A recent parabolic flight study reported dynamic changes in VS with reductions at the level of L3 when upright during hypergravity (∼1.8 *g*), which acutely increased during ∼20 s of microgravity (Swanenburg et al., 2020). Transient hypogravity (also induced by parabolic flight) reduced trunk admittance (a measure of trunk displacement as a function of external contact force), with apparent attenuation of para‐spinal but augmentation of deep abdominal muscle activity (De Martino et al., 2020). Interestingly, trunk exercise performance during ‘artificial gravity’ (with 1 *g* at the centre of mass) provided by short‐arm human centrifugation induced decreased thoracic VS, but with no change in cervical or upper/lower lumbar VS (Marcos‐Lorenzo et al., 2022) suggesting differential gravitational modulation of the vertebral column. Such modulation is likely to impact segmental spinal kinematics that may underlie gross spinal kinematic changes recently observed following long duration spaceflight (Bailey et al., 2022). However, given the challenges of in‐flight spinal investigation, ground‐based microgravity analogues are critical to evaluate pathophysiological mechanisms.

The most common microgravity ground‐based analogue is termed long duration head‐down tilt bed rest (HDTBR) (Hargens & Vico, 2016). However, during HDTBR typically participants are allowed to re‐orientate themselves and use a pillow in addition to performing up to 15 min of personal hygiene activities per day (Sundblad et al., 2014). Such activities that can generate loading upon the spinal column may contribute to the observation that spinal elongation during 24 h HDTBR is not significantly greater (∼1.2 cm) (Tyrrell et al., 1985) than 8 h of conventional horizontal sleep (Styf et al., 1997) although limited changes in trunk musculature and its activation are observed (De Martino et al., 2021).

Thus, HDTBR appears to be a relatively poor analogue of microgravity‐induced spinal column changes (Hargens & Vico, 2016) although recent stricter implementation of procedures such as prohibiting pillow use may affect spinal parameters in the future. Dry immersion (DI), where individuals lie on an impermeable membrane draped over a water‐filled tank (Navasiolava et al., 2011), is an alternative microgravity analogue which over a period of a couple of hours induces mild‐to‐moderate back pain; however, similar to HDTBR, increases in stature (spinal height) appear modest (∼1.3 cm) (Plehuna et al., 2022; Treffel et al., 2017). Muscle activation and forces associated with countering water motion and adoption of a relatively flexed position, with lumbar arching and head support out of the water during DI, may act to limit stature changes (Green & Scott, 2018).

Thus, a novel ground‐based analogue of spinal unloading was developed at King's College London termed hyper‐buoyancy flotation (HBF) (Unpublished Observations). HBF involves participants lying on a waterbed encased within an oversized frame which is partially (∼50%) filled with water supersaturated with magnesium sulfate (Epsom salts) to promote passive horizontal participant buoyancy. Eight hours of HBF elicits significant spinal elongation (2.2 ± 0.2 cm) and moderate reversible back pain (Unpublished Observations). Compared to DI, HBF has several advantages – both practically such as being amenable to instrumentation during floatation, and pragmatically allowing adoption of a passive non‐flexed position.

Furthermore, just 4 h of HBF also induces significant (1.8 ± 0.2 cm), albeit slightly lower, increments in stature along with moderate reversible back pain (Carvil, Russomano et al., 2017). Interestingly, HBF is also associated with lumbar kinematics modulation including motion sharing inequality (MSI) determined via quantitative fluoroscopy (Breen et al., 2023). This suggests HBF induces VS changes that may underlie vertebral kinematic modulation observed post‐flight (Bailey et al., 2022). MRI data from the HBF study also suggest unloading (and reloading) modulates lumbar lordosis in a manner not entirely dependent on IVD geometry changes.

Unfortunately, MRI, the gold‐standard for vertebral column imaging, is incompatible with spaceflight, whilst vertebral‐focused dual X‐ray absorptiometry (DXA) analysis (which could in principle be performed inflight) was inconclusive after 8 h HBF (Unpublished Observations). However, a novel in‐flight ultrasonic protocol developed by NASA (Marshburn et al., 2014) was employed for seven long‐duration mission astronauts, identifying 14 features of IVD pathology, including disc desiccation and osteophytes (Garcia et al., 2018). Yet, no significant changes in IVD height or angle were observed in‐flight, despite dynamic lumbar IVD geometry changes being reported in response to passive re‐orientation (Belavý et al., 2011), in addition to diurnal (Ledsome et al., 1996) and exercise‐induced loading (Kingsley et al., 2012) on Earth.

The failure to observe changes in orbit may relate to the performance of load‐bearing exercise in‐flight (Green & Scott, 2018). In fact, anecdotal reports suggest that intermittent axial loading and/or spinal flexion on the ISS can temporarily ameliorate back pain (Kerstman et al., 2012; Sayson et al., 2015). Furthermore, performance of squat exercise using the Advanced Resistive Exercise Device (ARED) has anecdotally been associated with acute reduction of microgravity‐induced stature increments (Green & Scott, 2018). Such uncontrolled dynamic, and thus potentially excessive, axial loading may contribute to post‐flight IVD pathology (Bailey et al., 2022) and herniation risk (Johnston et al., 2010). In fact, in‐flight axial loading has been provided by the Russian Pingvin (Penguin) suit that imposes (∼40 kg, i.e., ∼50% body weight (BW)) axial loading via bungee cords (Kozlovskaya et al., 1995; Severin et al., 1996).

However, the Pingvin suit was poorly tolerated (Waldie and Newman, 2011) and not employed as a specific spinal countermeasure (Green & Scott, 2018) even though axial‐loading garments have been suggested to promote spinal control on Earth (Rathinam et al., 2013). Graduated axial loading such as that provided by the Mk VI ‘SkinSuit’ which provides ∼0.2 *g* – comparable to a backpack mass shown to reduce stature on Earth via IVD compression (Neuschwander et al., 2010; Shymon et al., 2014) – has been shown to significantly ameliorate spinal elongation and back pain induced by 8 h HBF (Unpublished Observations) as well as restoring lumbar mobility and lordosis following 4 h HBF (Breen et al., 2023). Whilst compatible with ISS operations (Stabler et al., 2017) and the more severe constraints associated with exploration missions (Scott et al., 2019), the SkinSuit is not employed as a routine spinal countermeasure – in part due to the fact that the optimal axial loading ‘dose’ remains to be determined.

Supplementary (≥45% of the participant's BW) acute axial loading in 1 *g* has recently been shown to modulate spinal motor control (Glaus et al., 2021). Fifty percent BW additional (mass) loading significantly reduced spinal stiffness during standing (Häusler et al., 2020). Furthermore, 50% BW on a squat rack induced VS reductions (Häusler et al., 2020), with similar reductions when upright (Glaus et al., 2021). Hence, VS may be considered a proxy measurement of overall spinal motor control including passive elements.

Loaded, trunk flexion (e.g., 45° loaded seated flexion) has also been shown to induce IVD height reduction (Qin et al., 2022), with 30 s seated squat reducing cervical active VS (Hofstetter et al., 2021) on Earth. Thirty seconds of 50% BW may represent a similar axial ‘dose’ to that associated with an ISS (ARED) resistive exercise training session (Petersen et al., 2016). However, the effect of such axial loading on an ‘unloaded’ spine, and how it relates to 15 min of 1 *g* loading – equivalent to the most vulnerable spinal period post‐flight (Johnston et al., 2010) – is unknown.

Thus, the aim of the study was to determine whether 30 s seated 50% BW axial loading is comparable to 15 min sat in 1 *g* upon changes in stature, anterior lumbar IVD height (via ultrasound), passive VS and back pain induced by 4 h hyper‐buoyancy floatation (HBF) unloading.

## 2 METHODS

Sixteen (seven males) healthy recreationally active participants (age 23 ± 2 years, mass 70.7 ± 13.7 kg, height 172.7 ± 7.6 cm), conforming with current NASA astronaut anthropometric requirements (157–190 cm, 50–95 kg) , gave informed consent to participate in the study, which was approved by the King's College London ethics committee (HR‐18/19‐11548) and registered in 18/10/2022 at ClinicalTrials.org (NCT05590754). Prior to inclusion in the study, all subjects completed a medical screening to exclude current back/neck pain, musculoskeletal disorder, cardiovascular disease, spine surgery and being or suspected to be pregnant.

The study comprised two groups of eight participants upon whom identical measurements were obtained in both study locations: King's College London (London, UK) (four males, 25.5 ± 4.4 years, 63.5 ± 10.6 kg, 163.2 ± 5.6 cm) and the Instituto de Rehabilitación Funcional del Centro de Estudios Superiores in La Salle (Madrid, Spain) (three males, 27.0 ± 2.0 years, 68.4 ± 11.8 kg, 176.5 ± 10.9 cm). In each group participants were university students who were asked to attend the laboratory on two separate occasions on non‐sequential days following the initial medical screening and provision of written informed consent.

**FIGURE 1:**
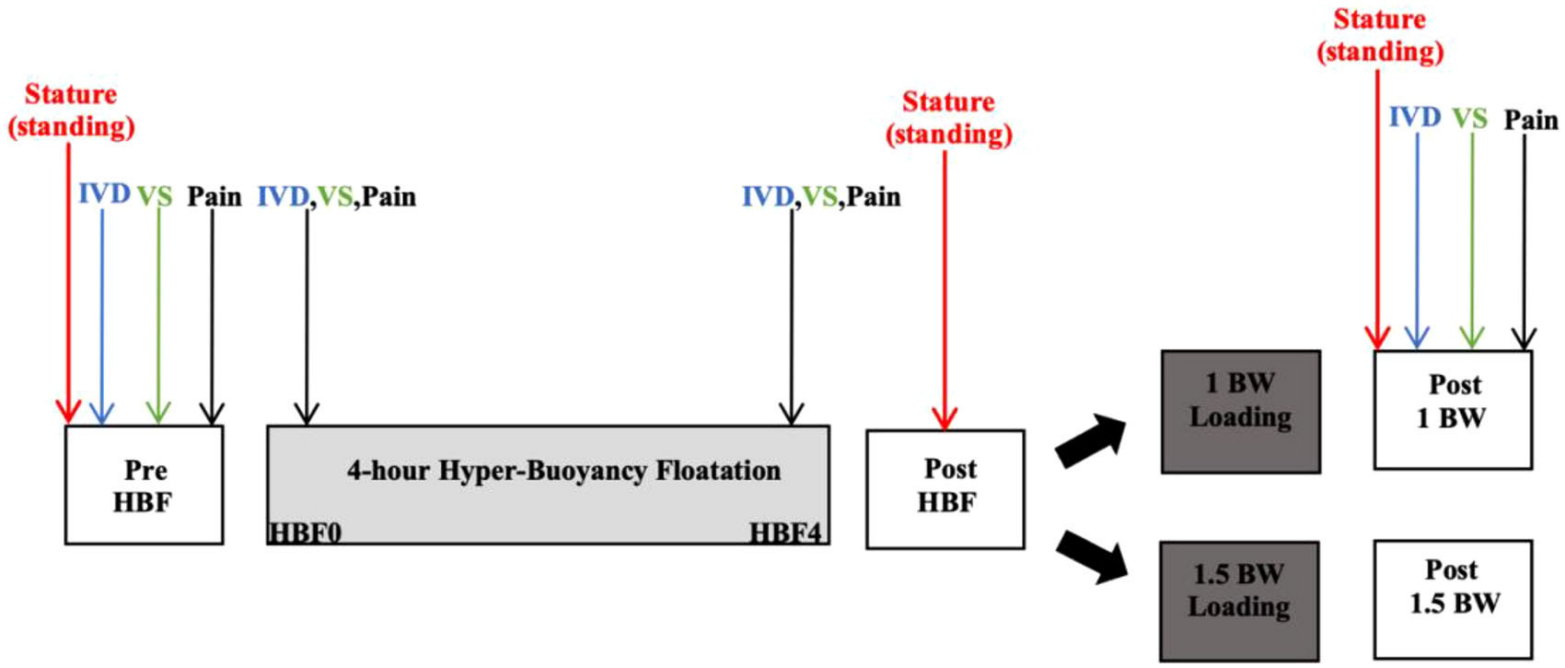
Experimental protocol. Standing stature, anterior lumbar IVD heights (L1 to S1; via ultrasound), vertebral stiffness (VS) and pain were determined prior to lying on the hyper‐buoyancy flotation (HBF) waterbed for 4 h (Pre HBF). Anterior lumbar IVD heights, VS and pain were also recorded immediately on lying on the bed (HBF0) and after 4 h of HBF (HBF4). Stature was measured immediately after HBF (PostHBF). Anterior IVD height, VS, and pain were re‐assessed in the 1 BW group (*n* = 8) after 15 min reloading in which participants sat upright throughout (Post 1 BW), whilst in the 1.5 BW group (*n* = 8) they were reassessed after performance of a 30 s seated squat with 50% of their body weight (Post 1.5 BW).

The first experimental session involved participant familiarization with the same HBF waterbed including passive re‐orientation (from supine to prone, and vice versa) and the relevant post‐HBF protocol. The second session involved employment of an identical 4 h HBF test protocol where in Madrid participants sat upright in a chair without back support for 15 min before post‐testing (1 BW), whilst in London participants performed a 30 s seated squat with 50% of their BW (1.5 BW) (Figure 1).

In the 1 BW group, the seated position supports only the axial load from gravity, causing the spinal muscles to counteract the effects of gravity. On the other hand, in the 1.5 BW group the countermeasure was specifically selected to target spinal muscles when the body position was at a 45° angle forward. This positioning was chosen to efficiently transmit the load through the spine, thus effectively activating the target and strengthening the spinal muscles (Häusler et al., 2020).

**FIGURE 2:**
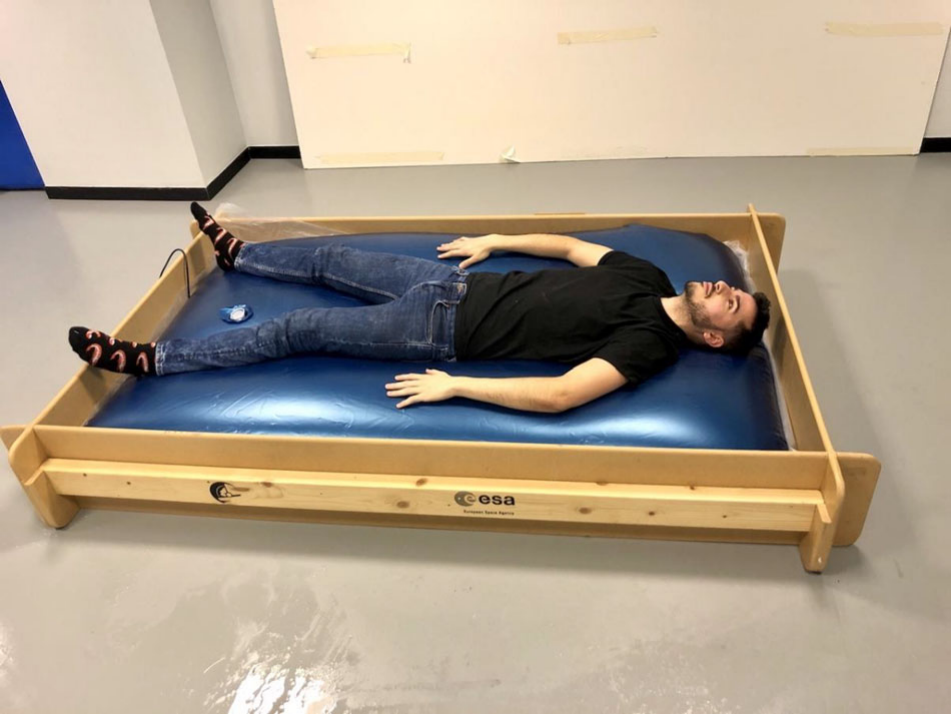
Participant lying supine on the hyper‐buoyancy flotation (HBF) waterbed. Participants lay in a position that ensured there was no contact with the wooden frame.

The HBF waterbed comprises a double waterbed (Figure 2) partially (∼50%) filled with water super‐saturated with magnesium sulfate (1.7 g cm^−3^), at a temperature between 34 and 36°C (regulated by an electronic ultra‐thin underbed heat pad), encased within a wooden frame. Participants moved gently onto the waterbed to allow the water to be displaced in a controlled manner so that they could quickly and smoothly adopt a resting supine position. Participants remained supine and motionless when they lay on the HBF except for temporary passive re‐orientation to and back from the prone position for VS assessment.

**FIGURE 3:**
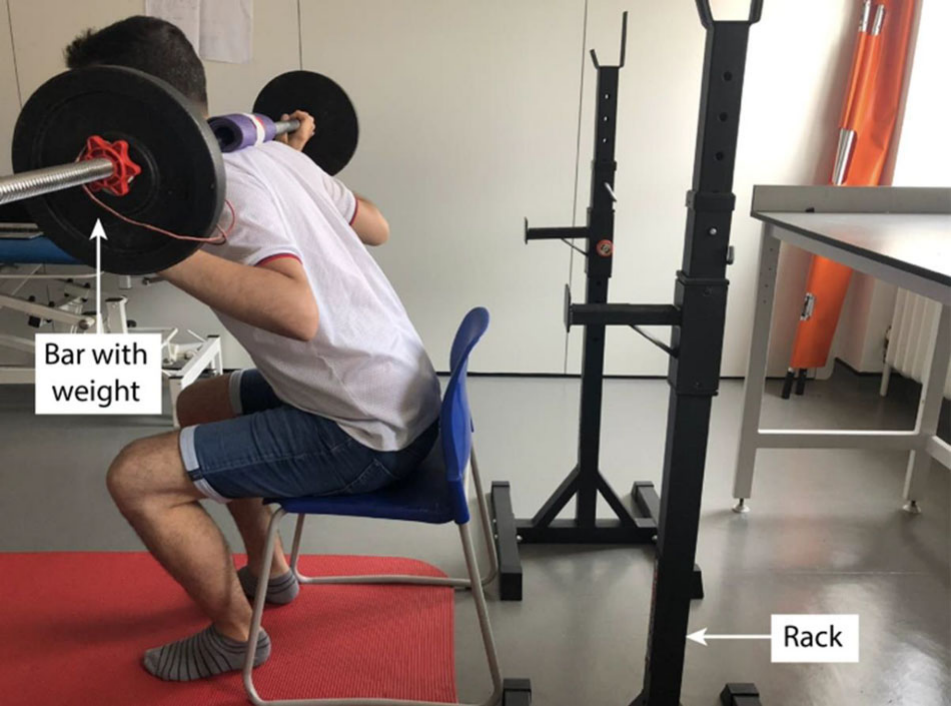
Static loading where the participant bore 50% of their body weight (BW) for 30s. The load is transmitted to the participant in a chair with back rest for safety reasons to control weight stabilization whilst seated and leaning forwards to 45° from vertical.

The 1.5 BW participants performed a 30 s 50% BW seated static load procedure, with a straight back and their feet facing forwards (greater than hip width apart). They were passed a pre‐weighted bar (loaded to 50% BW) from the rack behind them by the experimenter and an assistant standing on either side, after which they bore the weight on their shoulders and gently leaned forwards to 45° from vertical (Figure 3).

Participants were instructed to stabilize the weighted bar with their arms, but to bear the weight on their shoulders with a foam sheath fixed around the bar to distribute the load and thus reduce discomfort. Participants were advised to focus on maintaining stable hip flexion and thus allowing the lower (lumbar) vertebral column to take the strain. Supervision was provided throughout by the experimenter and an assistant standing on either side of the participant who simultaneously lifted the bar off the participant after 30 s and returned it to the rack.

Standing stature was determined with a commercially available stadiometer (Seca 217, Hamburg, Germany). Anterior lumbar IVD heights from L1 to S1 (L1–L2, L2–L3, L3–L4, L4–L5, L5–S1) were assessed via portable ultrasound (Lumify, Philips, Amsterdam, Netherlands) with a curvilinear array probe (8 MHz) connected to a Galaxy S2 tablet (Samsung, Seoul, South Korea). Ultrasonic measures were initially obtained when supine on a plinth, immediately on the HBF waterbed (HBF0), at 4 h of HBF (HBF4) and finally on a plinth following either 30s 50% BW loading (Post 1.5 BW) or after 15 min of 1 *g* (Post 1 BW) via portable ultrasound (Lumify, Philips) with a curvilinear array probe (8–15 MHz) connected to a Samsung Galaxy S2 tablet.

**FIGURE 4:**
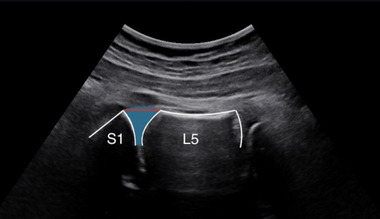
Long‐axis ultrasonic scanning L5–S1 anterior intervertebral disc height.

The ultrasonic images were acquired using a procedure based on that performed on the ISS (Marshburn et al., 2014) (Figure 4). Initial long‐axis views allowed clear visualization of the large abdominal vessels, aorta and inferior vena cava to assist location of acoustic windows for ultrasound imaging of IVDs. At least two longitudinal plane images per level were acquired (having optimized depth and focus) per time point, one with a high gain, and one with a low gain, to facilitate *post hoc* selection of the optimal image for anterior IVD height estimation. Anterior IVD height was determined as the distance from the most superior anterior point of the inferior vertebrae to the most inferior anterior point of the superior vertebrae at each lumbar IVD level. Ultrasound is considered a reliable and accurate methodology to measure anterior disc height distances with a standard error of 0.01–0.02 and a mean intraclass correlation coefficient (ICC) of 0.98–1.00 (Sobczak et al., 2016). In our study all images were collected by the same ultrasonographer and analysed offline.

**FIGURE 5:**
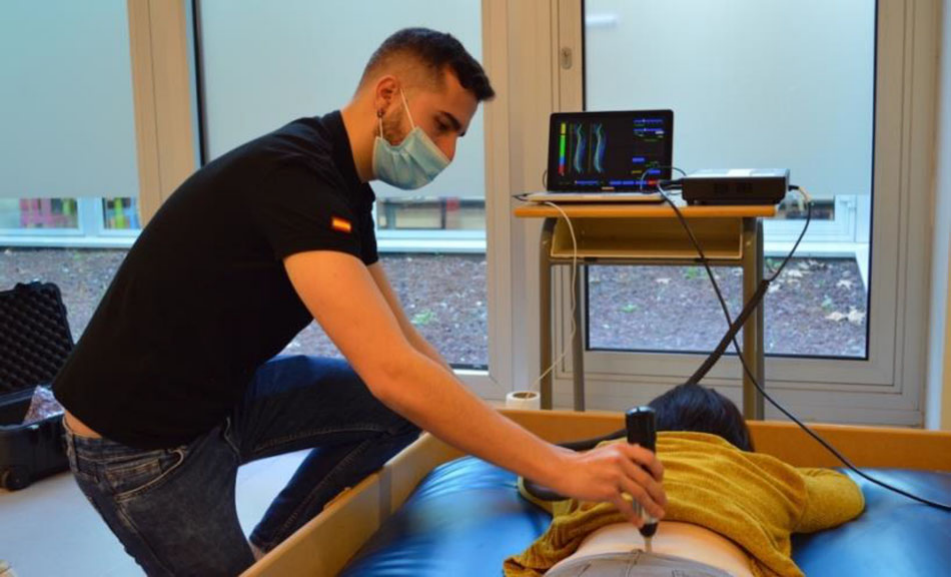
Passive vertebral stiffness acquisition using the handheld differential vertebral stiffness transducer (PulStar, Sense Technology Inc., Murrysville, PA, USA) during HBF induced unloading.

Passive VS from C1 to L5 was acquired in the prone position pre‐HBF (on a plinth) immediately when lying on the HBF waterbed (HBF0) and after 4 h of HBF (HBF4) and finally back on the plinth after either 30 s 50% BW loading (Post 1.5 BW) or after 15 min of 1 *g* (Post 1 BW). Passive VS was estimated with a handheld differential VS transducer (PulStar, Sense Technology Inc., Murrysville, PA, USA) manually placed and held perpendicularly to the surface of the back upon each vertebral spinous process by the same evaluator (Hofstetter et al., 2018; Leach et al., 2003). A preload >18 N was required to trigger instigation of a 27 N impulse which compresses the soft tissue between the transducer head and the target spinous process (Figure 5).

The propagation properties of the triggered impulse reflect VS (Girod et al., 1997) captured by a load cell (N) in the transducer head and automatically stored in dedicated software (PulStarFRAS, Sense Technology). PulStar VS recordings (including C1–L5) are reported to possess good test–retest reliability (Hofstetter et al., 2018) and have been shown to be reliable with ICCs of 0.65–0.99 (Hofstetter et al., 2018), being excellent across the spine, with ICC > 0.79 and Cronbach's α 0.80–0.948 (Hofstetter et al., 2021).

Back pain ratings were acquired using the CHAPS back pain rating scale used in previous HBF studies (Carvil, Russomano et al., 2017). The CHAPS back pain scale employs integers from 0 (no observable pain) to 10, with 10 corresponding to pain that it is not possible to withstand. To minimize participant movement, verbal declarations of back pain ratings were noted by an experimenter.

### 2.1 Data analysis

All data were normally distributed (Shapiro–Wilk test). Participant age, height and body mass prior to testing were compared between groups (1 BW and 1.5 BW) by Student's unpaired *t*‐test.

Stature was determined prior to HBF (Pre HBF), immediately after 4 h of HBF (Post HBF) and after 15 min when sitting upright (Post 1 BW) and immediately after 30 s 50% BW additional static loading (Post 1.5 BW). Anterior lumbar IVD heights, stiffness at each vertebra, and back pain were recorded immediately upon lying on the HBF (HBF0), after 4 h HBF (HBF4), in addition to immediately after 15 min sitting upright in the 1 *g* (Post 1 BW) and static loading (Post 1.5 BW) conditions. Passive VS values were averaged across the entire column (C1–L5), and for the cervical (C1–C7), thoracic (T1–T10), upper (T11–L2) and lower lumbar (L3–L5) regions.

Test–retest reliability of stature, anterior disc height and passive VS were assessed via calculation of mean ICC and Cronbach's α value (HBF0 vs. HBF4) from stature, anterior disc height (L2–S1) and entire column VS, classified according to standard guidelines (Bobak et al., 2018).

As initial stature (Pre HBF) differed between groups, the effect of unloading (Δ HBF; Post HBF − Pre HBF in each group) and reloading (1 BW, Δ Post 1 BW − Post HBF; and 1.5 BW, Δ Post 1.5 BW − Post HBF) was assessed via a two‐way (2 × 2) ANCOVA with body mass as a co‐variate.

The effect of HBF‐induced unloading (Δ HBF; HBF0, HBF4) and reloading (1 BW, Δ Post 1 BW − HBF4; and 1.5 BW, Δ Post 1.5 BW − HBF4) and overall group (GROUP) upon lumbar IVD (L1–S1) anterior disc heights, VS (entire column, cervical, thoracic, upper and lumbar regions) and back pain were assessed via a two‐way ANCOVA with body mass as a co‐variate. Having established that the HBF unloading effect did not differ between groups, the effect of re‐loading (1 BW vs. 1.5 BW) following HBF was compared with an unpaired *t*‐test between Δ Post BW − HBF and Δ Post 1.5 BW − HBF4.

Pearson's correlation coefficient (*r*) was calculated to assess the relationship between ∆ back pain and ∆ standing stature, and with ∆ anterior IVD height (L1–S1) and ∆ Entire Column, Cervical, Thoracic, Upper Lumbar and Lower Lumbar VS during HBF unloading (Δ HBF, HBF4 − HBF0) and reloading (1 BW, Δ Post 1 BW − Post HBF; and 1.5 BW, Δ Post 1.5 BW − Post HBF). Pearson's correlation coefficient (*r*) was also calculated to evaluate the relationship between ∆ standing stature with ∆ anterior IVD height (L1–S1) and ∆ Entire Column, Cervical, Thoracic, Upper Lumbar and Lower Lumbar VS during HBF unloading (Δ HBF, HBF4 − HBF0) and reloading (1 BW, Δ Post 1 BW − Post HBF; and 1.5 BW, Δ Post 1.5 BW − Post HBF).

All data are reported as means ± standard deviation (SD) with percentage change and associated 95% confidence intervals (CI) provided where appropriate. Statistical tests were performed using IBM SPSS version 21 (IBM Corp., Armonk, NY, USA) with *P* < 0.05 considered to indicate statistical significance.

Based on the mean and standard deviation of stature changes (primary outcome measure) induced by 8 h unloading in a previous study (Styf et al., 1997), an online sample size calculation was performed (G*Power statistical software; University of Düsseldorf, Germany) yielding a total required sample of 16, assuming a medium effect size of 0.6, an α of 0.05 and a power of 1 − β = 0.8.

## 3 RESULTS

All 16 participants completed 4 h HBF, and subsequent 15 min seated 1 *g* exposure (1 BW) or 30 s static 50% BW loading (1.5 BW) without issue. Age and body mass did not significantly differ between groups.

Test–retest evaluation, comparing HBF0 and HBF4, showed stature measures to possess excellent reliability (ICC = 1.00; Cronbach's α = 1.00). IVD image quality was satisfactory except for L1–L2, which is not reported. Reliability for L5–S1 anterior disc height was medium (ICC = 0.51; α = 0.68); for L3–L4 was good (ICC = 0.62; α = 0.76); for L2–L3 was medium (ICC = 0.4; α = 0.60). Entire column VS (ICC = 0.59; α = 0.74) reliability was good.

### 3.1 Standing stature

Four hours of HBF unloading and reloading significantly modulated standing stature [*F* (1, 32) = 272.951, *P *< 0.001], with no group [*F* (1,32) = 1.580, *P* = 0.220] or interaction effect [*F* (1, 32) = 2.625, *P* = 0.117] as similar stature increments were induced in 1 BW (+1.0%) and 1.5 BW (+1.1%) groups (Table 1). On average 4 h HBF across the two groups induced a 1.55 ± 0.50 cm (∼1.0%) increase in stature with increments observed in all individuals.

**TABLE 1:** Mean ± SD delta (∆) standing stature (cm) (and percentage change) and 95% CI induced by 4 h of hyper‐buoyancy floatation (HBF) unloading (Δ HBF; Post HBF − Pre HBF) in the 1 BW group (*n* = 8) and 1.5 BW group (*n* = 8), and then immediately after participants performed a 30 s seated squat with 50% of their BW (Δ Post 1.5 BW − Post HBF) in the 1.5 BW group, or 15 min 1 *g* reloading where participants sat upright throughout (Δ Post 1 BW − Post HBF) in the 1 BW group.

	∆ 4 h HBF		∆ Post axial reloading	
	1 BW	1.5 BW	1 BW	1.5 BW
Standing stature (cm)	+1.32 ± 0.54 (+1.00 ± 0.30%)	+1.78 ± 0.36 (+1.09 ± 0.21%)	−0.72 ± 0.25 (−0.40 ± 0.15%)	−0.71 ± 0.35 (−0.44 ± 0.22%)
95% CI	0.86, 1.78	1.47, 2.08	−0.93, −0.51	−1.00, −0.41

Standing stature increments induced by 4 h HBF were similarly attenuated – but not entirely reversed – by axial reloading in both the 1 BW and 1.5 BW groups, with no difference (*P* = 0.595) in 1 BW group vs 1.5 BW post axial reloading (Table 1). On average, axial reloading across the two groups reduced stature by 0.71 ± 0.29 cm (∼40%).

### 3.2 Anterior IVD height

Four hours of HBF‐induced unloading and reloading significantly modulated L2–L3 [*F* (1, 32) = 11.861, *P* = 0.002], L3–L4 [*F* (1, 32) = 25.814, *P *< 0.001], L4–L5 [*F* (1, 32) = 7.025, *P* = 0.013] and L5–S1 [*F* (1, 32) = 23.735, *P *< 0.001] anterior IVD disc height (Table 2). However, no group or interaction effect was observed at any level. Thus, on average 4 h HBF across the two groups induced increases of IVD disc height at L2–L3 (0.12 ± 0.09 cm; 19 ± 17%), L3–L4 (0.12 ± 0.10 cm; 16 ± 13%), L4–L5 (0.14 ± 0.13 cm; 18 ± 14%) and L5–S1 (0.20 ± 0.22 cm; 12 ± 10%).

**TABLE 2:** Mean ± SD delta (∆) anterior intervertebral disc (IVD) (L2–S1) height (cm) (and percentage change) and associated 95% CI induced by 4 h of hyper‐buoyancy floatation (HBF) unloading (Δ HBF; HBF4 − HBF0) in the 1 BW group (*n* = 8) and 1.5 BW group (*n* = 8), and then immediately after participants performed a 30 s seated squat with 50% of their BW (Δ Post 1.5 BW − HBF4) in the 1.5 BW group, or 15 min 1 *g* reloading where participants sat upright throughout (Δ Post 1 BW − HBF4) in the 1 BW group.

	Anterior IVD height (cm)			
	∆ 4 h HBF		∆ Post axial reloading	
	1 BW	1.5 BW	1 BW	1.5 BW
L2–L3	0.13 ± 0.10 (19 ± 11%)	0.11 ± 0.08 (20 ± 24%)	−0.01 ± 0.10 (−2 ± 20%)	−0.01 ± 0.12 (−3 ± 18%)
95% CI	0.04, 0.21	0.03, 0.18	−0.12, 0.09	−0.12, 0.08
L3–L4	0.16 ± 0.09 (21 ± 8%)	0.08 ± 0.09 (12 ± 19%)	−0.08 ± 0.09 (−10 ± 13%)	−0.04 ± 0.12 (−6 ± 20%)
95% CI	0.08, 0.24	0.01, 0.16	−0.18, 0.01	−0.14, 0.05
L4–L5	0.10 ± 0.12 (10 ± 12%)	0.18 ± 0.14 (26 ± 16%)	−0.01 ± 0.24 (−1 ± 22%)	−0.03 ± 0.17 (−5 ± 22%)
95% CI	−0.01, 0.20	0.06, 0.30	−0.20, 0.19	−0.18, 0.10
L5–S1	0.11 ± 0.04 (10 ± 3%)	0.29 ± 0.29 (14 ± 18%)	−0.09 ± 0.14 (−8 ± 12%)	−0.19 ± 0.21 (−17 ± 19%)
95% CI	0.08, 0.15	0.04, 0.53	−0.21, 0.29	−0.37, −0.01

Increments in anterior IVD heights induced by 4 h HBF were significantly attenuated – but not entirely reversed – at all levels by axial reloading in both the 1 and 1.5 BW groups (Table 2). On average axial reloading across the two groups reduced L2–L3 (−0.01 ± 0.11 cm; −2 ± 19%), L3–L4 (−0.06 ± 0.11 cm; −8 ± 13%), L4–L5 (−0.02 ± 0.20 cm; −3 ± 22%) and L5–S1 (−0.14 ± 0.18 cm; −12 ± 15%). However, no significant differences were observed (L2–L3, *P* = 0.80; L3–L4, *P* = 0.69; L4–L5, *P* = 0.71; L5–S1, *P* = 0.72) between the 1 BW group and 1.5 BW groups post‐reloading.

### 3.3 Passive VS

Four hours of HBF‐induced unloading and reloading induced no significant change in passive VS across the entire column [*F* (1, 32) = 1.259, *P* = 0.272], cervical [*F* (1, 32) = 3.312, *P* = 0.080], thoracic [*F* (1, 32) = 0.565, *P* = 0.459], upper lumbar [*F* (1, 32) = 0.001, *P* = 0.986] or lower lumbar [*F* (1, 32) = 0.779, *P* = 0.385] regions (Table 3). No group or interaction effect was observed at any level. However, on average 4 h HBF across the two groups tended to reduce VS of the entire column (−2.02 ± 3.53 N; −3 ± 5%), cervical (−1.60 ± 3.37 N; −3 ± 5%), thoracic (−1.80 ± 4.58 N; −3 ± 6%), and upper (−3.08 ± 4.33 N; −5 ± 7%) and lower (−2.22 ± 5.21 N; −4 ± 9%) lumbar regions.

**TABLE 3:** Mean ± SD delta (∆) passive vertebral stiffness (VS; newtons) change of the entire column (C1–L5) (and percentage change) and associated 95% CI, cervical, thoracic and lumbar segments induced by 4 h of hyper‐buoyancy floatation (HBF) unloading (Δ HBF; HBF4 − HBF0) in the 1 BW group (*n* = 8) and 1.5 BW group (*n* = 8), and then immediately after participants performed a 30 s seated squat with 50% of their BW (Δ Post 1.5 BW − HBF4) in the 1.5 BW group, or 15 min 1 *g* reloading where participants sat upright throughout (Δ Post 1 BW − HBF4) in the 1 BW group.

	Passive vertebral stiffness (N)			
	∆ 4 h HBF		∆ Post axial reloading	
	1 BW	1.5 BW	1 BW	1.5 BW
Entire column	−2.11 ± 2.15 (−3 ± 2%)	−1.93 ± 4.70 (−3 ± 8%)	−3.68 ± 3.03 (−6 ± 4%)	−3.34 ± 4.44 (−6 ± 6%)
95% CI	−0.30, −5.86	1.99, −6.21	−0.30, −5.86	1.99, −6.21
Cervical	−1.95 ± 1.87 (−3 ± 2%)	−1.26 ± 4.53 (−2 ± 8%)	−3.89 ± 2.54 (−6 ± 3%)	−4.71 ± 6.11 (−8 ± 12%)
95% CI	−0.38, −5.05	2.53, −6.01	−0.38, −5.05	2.53, −6.01
Thoracic	−2.68 ± 4.31 (−4 ± 4%)	−0.92 ± 4.95 (−2 ± 8%)	−2.31 ± 4.34 (−3 ± 5%)	−3.59 ± 3.43 (−6 ± 7%)
95% CI	−6.29, 0.93	−5.07, 3.21	−6.29, 0.93	−5.07, 3.21
Upper lumbar	−1.76 ± 2.39 (−3 ± 5%)	−4.40 ± 5.52 (−8 ± 9%)	−4.55 ± 5.97 (−7 ± 10%)	−1.68 ± 5.36 (−3 ± 11%)
95% CI	−3.76, 0.24	−9.03, 0.21	−3.76, 0.24	−9.03, 0.21
Lower lumbar	−0.89 ± 4.41 (−2 ± 8%)	−6.74 ± 5.79 (−10 ± 12%)	−0.89 ± 4.41 (−2 ± 8%)	−6.74 ± 5.79 (−10 ± 12%)
95% CI	−4.58, 2.79	−8.47, 1.37	−4.58, 2.79	−8.47, 1.37

Axial reloading had no significant effect on VS in any region (Table 3), with no significant differences observed (entire column, *P* = 0.863; cervical, *P* = 0.730; thoracic, *P* = 0.526; upper lumbar, *P* = 0.329; and lower lumbar, *P* = 0.155 regions) between the 1 BW and 1.5 BW groups. On average reloading across the two groups tended to reduce VS of the entire column (−3.51 ± 3.70; −6 ± 5%), cervical (−4.30 ± 4.32; −7 ± 7%), thoracic (−2.95 ± 3.88; −4 ± 6%), and upper (−3.11 ± 5.66; −5 ± 10%) and lower (−4.14 ± 6.81; −6 ± 13%) lumbar regions.

### 3.4 Back pain

All participants completed a medical screening to exclude current back/neck pain, and therefore none reported pain prior to HBF, although all individuals reported some back pain during HBF unloading. No individual participant reported severe back pain (i.e., greater than 4.5/10) during HBF or subsequent axial reloading. Four hours of HBF and reloading modulated [*F* (1,32) = 132.914, *P *< 0.001] back pain, in both the 1 BW and 1.5 BW groups, with no group or interaction effect (Table 4). On average 4 h HBF across the two groups induced moderate back pain ratings of 2.90 ± 1.26 (28 ± 12%).

**TABLE 4:** Mean ± SD delta (∆) back pain (and percentage change) and associated 95% CI induced by 4 h of hyper‐buoyancy floatation (HBF) unloading (Δ HBF; HBF4 − Pre HBF) in the 1 BW group (*n* = 8) and 1.5 BW group (*n* = 8), and then immediately after participants performed a 30s seated squat with 50% of their BW (Δ Post 1.5 BW − HBF4) in the 1.5 BW group, or 15 min 1 *g* reloading where participants sat upright throughout (Δ Post 1 BW − HBF4) in the 1 BW group.

	∆ 4 h HBF		∆ Post axial reloading	
	1 BW	1.5 BW	1 BW	1.5 BW
Back pain (0/10)	+2.75 ± 1.41 (+27 ± 14%)	+3.06 ± 1.17 (+30 ± 11%)	−2.37 ± 0.51 (−86 ± 15%)	−2.68 ± 0.74 (−88 ± 18%)
95% CI	1.56, 3.93	2.07, 4.04	−3.69, −1.05	−3.53, −1.85

Back pain induced by 4 h HBF was significantly attenuated in both 1 BW (−2.37; −86%) and 1.5 BW (−2.68; −88%), with no difference between post axial reloading groups (*P* = 1.000). On average axial reloading across the two groups reduced back pain by 2.53 ± 1.28 (−87 ± 16%).

### 3.5 Correlations

Pooled HBF‐induced and axial reloading positively correlated with Δ L2–L3, Δ L4–L5 and Δ L5–S1 anterior IVD height and Δ back pain. Δ stature did not statistically correlate with Δ overall passive VS, or that of any segment (Table 5).

**TABLE 5:** Pearson's correlation (*r*) of stature increase induced by 4 h of hyper‐buoyancy floatation (HBF) unloading (*n* = 16) (Δ HBF; HBF4 − HBF0) and Δ post axial reloading (post axial load − Post HBF) anterior disc height (L2–S1) and (post − pre HBF) entire column, cervical, thoracic, upper lumbar and lower lumbar vertebral stiffness and back pain (Δ HBF; HBF4 − HBF0). *Significant difference, *P* < 0.05.

Variable	L2–L3	L3–L4	L4–L5	L5–S1	Entire column	Cervical	Thoracic	Upper lumbar	Lower lumbar	Pain
*R*	0.38	0.61	0.39	0.64	0.20	0.34	0.15	−0.03	0.12	0.89
*P*	*0.03	*0.01	*0.02	*0.01	0.25	0.06	0.39	0.86	0.48	*0.01

Δ Back pain also positively correlated with Δ L2–L3, Δ L3–L4 and Δ L5–S1 anterior IVD height, but Δ L4–L5 IVD height failed to reach statistical significance. Δ back pain also failed to correlate with Δ overall passive VS, or that of any segment (Table 6).

**TABLE 6:** Pearson correlation (*r*) of back pain induced by 4 h of hyper‐buoyancy floatation (HBF) unloading (*n* = 16) (Δ HBF; HBF4 − HBF0) and Δ post axial reloading (post axial load − post HBF) standing stature, Δ (post axial reloading − HBF4), anterior disc height (L2–S1) and (post‐pre HBF) entire column, cervical, thoracic, upper lumbar and lower lumbar vertebral stiffness. *Significant difference, *P* < 0.05.

Variable	Stature	L2–L3	L3–L4	L4–L5	L5–S1	Entire column	Cervical	Thoracic	Upper lumbar	Lower lumbar
*r*	0.89	0.482	0.70	0.31	0.56	0.23	0.31	0.20	−0.02	0.19
*P*	*0.01	*0.05	*0.01	0.08	*0.01	0.19	0.07	0.27	0.87	0.29

## 4 DISCUSSION

The main findings of the study were that 4 h HBF unloading induced significant increments in standing stature, back pain and anterior IVD height at all levels from L2 to S1 in both groups. Stature, back pain and anterior IVD height increments and induced back pain were similarly (albeit neither entirely) attenuated by 15 min upright seating and 30 s 50% BW axial loading. In contrast, passive VS measures were unchanged by 4 h HBF or axial re‐loading. HBF‐induced stature positively correlated with increased L2–L3, L3–L4, L4–L5, L5–S1 and back pain. Back pain also positively correlated with increased stature and L2–L3, L3–L4, L5–S1 and IVD height.

### 4.1 Standing stature

Participants’ standing stature, age and weight did not differ between groups, despite the UK (i.e., 1.5 BW group) being on average taller than the Spanish (i.e., 1 BW group) population (NCD Risk Factor Collaboration (NCD‐RisC), 2016). All participants in both the 1 and 1.5 BW groups experienced significant stature elongation during HBF of ∼1.6 ± 0.5 cm equating to ∼+1%. Such stature increments are similar to those previously observed after 4 h HBF in the UK by Carvil and co‐workers (1.80 ± 0.22 cm) (Carvil, Russomano et al., 2017) and in Spain (1.60 ± 0.52 cm) (Unpublished Observations). However, 4 h HBF stature changes are lower than those observed after 8 h HBF in the day (2.2 ± 0.2 cm) (Unpublished Observations) and overnight (2.06 ± 0.4 cm) (Breen et al., 2023).

Nevertheless, 4 h HBF stature increments are in excess of those observed in response to 24 h HDTBR (∼1.2 cm) (Tyrrell et al., 1985) and 3 days of DI (∼1.3 cm) (Plehuna et al., 2022). However, increments remain less than those reported prior to the ISS (Brown, 1977) or the 3% recently reported early in‐flight that remain relatively stable throughout the mission (Young et al., 2021). Thus, whilst HBF may not fully replicate microgravity, it does appear to induce rapid stature increments. Furthermore, in common with spaceflight, there is inter‐individual variation, although, despite significant stature differences in initial stature (Pre HBF), increments were similar in the 1 BW group (2.1 cm; +1.1%) and the 1.5 BW group (2.3 cm; +1.1%). Thus, HBF may be of utility operationally to predict microgravity‐induced elongation for the sizing of spacesuits.

Thirty seconds of 50% BW static load was well‐tolerated and provided amelioration of HBF‐induced stature increments similar to 15 min 1 *g* loading (∼40%). Such amelioration is comparable to that reported by the Mk VI SkinSuit, which provides ∼0.2 *g* axial loading (Breen et al., 2023), and by acute (10–30% BW) backpack wear when upright in a 1 *g* environment (Neuschwander et al., 2010; Shymon et al., 2014). It is also consistent with anecdotal reports of stature reductions induced by the use of ARED – potentially via IVD compression (Green & Scott, 2018). Thus, our findings extend those of Qin and co‐workers (Qin et al., 2022) and suggest that even brief axial loading generated with a relative low BW in 30 s induces stature responses similar to 15 min in 1 *g*.

### 4.2 Lumbar IVD height

We observed satisfactory ultrasonic images across the lumbar spine (L2–S1) but not L1–L2 – due to superposition of abdominal viscera, similar to previous studies (Marcos‐Lorenzo et al., 2022). Four hours of HBF‐induced unloading significantly increased anterior IVD height from L2–L3 to L5–S1 with no difference between groups. Increases in IVD height were substantial ranging from 12 ± 10% at L5–S1 to 19 ± 17% at L2–L3 – which contrasts with the failure to observe IVD height changes following the same methodology in orbit although disc desiccation and osteophytes were reported (Garcia et al., 2018) consistent with post‐flight MRI (Bailey et al., 2022; Chang et al., 2016). Also reported were no change in IVD angle despite microgravity being associated with IVD swelling (Sayson & Hargens, 2008) and post‐flight spinal endplate irregularities (Bailey et al., 2022), although it may also relate to repeated exposure to in‐flight load‐bearing exercise – including ARED and T2 treadmill (Green & Scott, 2018).

Increments in IVD height induced by 4 h HBF were significantly attenuated – but not entirely reversed – similarly at all levels in both the 1 BW and 1.5 BW groups. Reductions in IVD height augmentation ranged from −12% at L5–S1 to −2% at L2–L3 consistent with weight sharing through the column. The fact that similar effects were observed in both groups suggests that acute loaded trunk flexion is likely to induce significant IVD height reduction in microgravity, as has been reported on Earth (Qin et al., 2022). It also suggests that the ARED stature reductions may involve IVD compression – particularly as the loading is more dynamic than in our study – which may be advantageous in the short term, but may contribute to post‐flight IVD pathology (Bailey et al., 2022) and herniation risk (Johnston et al., 2010).

The fact that ultrasound was sensitive to such changes supports its use to assess dynamic IVD height changes in‐flight including following ARED use, along with candidate countermeasures such as short‐arm human centrifugation where IVD compression using the same ultrasound methodology has been observed following trunk exercises (lateral stabilization, trunk rotation, and isometric abdominal) (Marcos‐Lorenzo et al., 2022).

Thus, further evaluation is critical to inform whether periodic moderate (∼0.2 *g*) static axial loading such as that provided by Mk VI SkinSuit (Unpublished Observations), the 0.7 *g* reported when donning the Mk III Gravity Loading Countermeasure SkinSuit (GLCS) (Carvil, Attias et al., 2017), or the impulse loading associated with ARED use are actually protective against microgravity‐induced spinal pathology (Green & Scott, 2018).

In our study we observed good IVD height reliability with an ICC of 0.40–0.62 across the lumbar spinal column consistent with that reported clinically (Sobczak et al., 2016). However, achieving and maintaining probe placement is very challenging in microgravity despite the use of the crew restraint system, as crew are not fully trained sonographers – which may limit sensitivity.

Thus, whilst ultrasonic assessment of IVD height and IVD angle with HBF is warranted as spinal curvature flattening is a potential mechanism underlying stature increments (Andreoni et al., 2000), whether similar results can be obtained in microgravity is unclear.

### 4.3 Passive VS

Four hours of HBF induced no significant change in passive VS across the entire column, nor at any evaluated spinal segment (cervical, thoracic, upper and lower lumbar) in either group. This contrasts with our hypothesis that HBF‐induced spinal length changes would elicit significant modulation of spinal stabilization – reflected in a reduction in passive VS. For instance, an increase of active VS has been reported in response to acute (<22 s) microgravity – although it should be acknowledged that immediately follows a brief (<5 s) period of hypergravity (∼1.8 *g*) (Swanenburg et al., 2020). The failure to observe any changes is also surprising given that Mk VI SkinSuit reloading (0.2g) has been shown to increase lumbar mobility (presumably maintaining VS) following HBF, compared to HBF alone (Breen et al., 2023). However, it should be noted that the effect of HBF per se was not determined.

In our study, the entire column passive VS tended to be reduced (−3 ± 5%) during HBF unloading. Similar trends were noted in cervical passive VS (−3 ± 5%) presumably related to participants being required to remain motionless with their head resting neutrally on the HBF bed. Thoracic passive VS also was attenuated despite the segment possessing other elements that can contribute to stabilization (i.e., stiffness) such as the sternum and rib cage, which provide additional stiffness and are thus less sensitive to modulations in spinal stiffness during axial loading (Hofstetter et al., 2018).

Whilst also non‐statistically significant, slightly stronger reductive treads were noted in the upper (−5 ± 7%) and lower lumbar (−4 ± 9%) segment. Interestingly, the lower lumbar segment is typically most vulnerable to spinal dysfunction post long duration spaceflight. In fact, loss of lumbar curvature (Andreoni et al., 2000), trunk muscle atrophy (Burkhart et al., 2019; Chang et al., 2016) and evidence of vertebral dysfunction (Hides et al., 2021) including impaired spinal kinematics and modulation of spinal stiffness (Bailey et al., 2022) have been reported that may relate to reduced para‐spinal muscle tone (McNamara et al., 2019).

Unexpectedly, neither 15 min of 1 *g* exposure nor 30 s 50% BW static load induced significant modulation of passive VS from that following 4 h HBF unloading. In fact, passive VS tended to be lower across the entire column and each segment than immediately following HBF. Interestingly, the segment with the greatest tendency to fall was the lower lumbar. Whilst non‐significant the trend is consistent with reductions in active VS during axial loading associated with 0%, 10%, 45% and 80% of BW (Glaus et al., 2021) or ≥45% axial mass loading when upright (Glaus et al., 2021), acute exposure to hypergravity (∼1.8 *g*) in a parabolic flight (Swanenburg et al., 2023, 2020), and with squat loading in 1 *g* (Häusler et al., 2020).

In fact, whilst we observed a decrease in active VS on a short‐arm human centrifuge 1 *g* at CoM with concurrent trunk exercise performance, no change in passive VS was evident (Marcos‐Lorenzo et al., 2022). This is consistent with VS being highly posture‐dependent (Häusler et al., 2020). Indeed active VS is affected by connective tissue characteristics (Stokes & Gardner‐Morse, 2003) and active motor control (Hodges et al., 2013), which renders it more sensitive measure to change (Glaus et al., 2021). Thus, further studies evaluating active VS, pre and post HBF following axial loading with larger sample sizes are warranted, as whilst VS assessed by the PulStar has been shown to possess good test–retest reliability (Hofstetter et al., 2018) and reproducibility (Hofstetter et al., 2021), significant (∼10–15%) inter‐individual variability is observed (Häusler et al., 2020).

### 4.4 Back pain

Four hours of HBF induced progressive mild back pain of similar magnitude and time course in both groups (mean 2.9 ± 1.3). Whilst all participants experienced back pain during HBF, in none was pain excessive, with 4.5/10 being the highest reported. Participants most often reported lower back pain, although pain in the base of the neck was also noted. The induced back pain is consistent with that reported in other HBF studies (Breen et al., 2023; Carvil, Russomano et al., 2017). Such reports are broadly consistent with those in microgravity where back pain is transient (developing in the first few hours (i.e., <8 h) of free floating), moderate (<5/10) and presents predominantly in the lumbar region, although thoracic and cervical pain is reported (Kerstman et al., 2012; Pool‐Goudzwaard et al., 2015; Wing et al., 1991).

As such, HBF appears to be superior to HDTBR (Belavý et al., 2008) and DI (Plehuna et al., 2022; Treffel et al., 2017) in terms of validity as an analogue of microgravity‐induced back pain. However, confirmation of whether HBF‐induced back pain is similar in location, magnitude and time course to space‐adaptation back pain (Penchev et al., 2021) is warranted, although this is challenging as experimental evaluation is not typically performed in the first 4 days of missions.

Interestingly, back pain was largely, albeit not entirely, ameliorated by 15 min of 1 *g* (−86%) or 30 s 50% BW (−87%), which is in excess of that observed in HBF studies involving Mk VI SkinSuit wear (Breen et al., 2023; Carvil, Russomano et al., 2017). However, most participants had no residual back pain. This finding is consistent with reports that spinal flexion (Sayson et al., 2015) or ‘fetal tucks’ can reduce back pain in space (Kerstman et al., 2012) although it suggests that space adaptation back pain is unlikely to be discogenic, as such pain on Earth is reported to worsen with flexion (McKenzie, 2003). It should, however, be noted that the longer‐term consequences of such manoeuvres on the spinal column may be less advantageous including potentially contributing to post‐flight issues such as IVD herniation (Johnston et al., 2010). Thus, as at the ISS there are no formal spinal‐focused countermeasures, with back pain management limited to conventional pharmacological pain approaches (Wotring, 2015), further HBF studies are warranted to optimise inflight approaches.

### 4.5 Correlations

HBF‐reloading induced stature changes positively correlated with induced back pain – in agreement with that reported for the lumbar segment when not wearing the Mk VI SkinSuit in a previous (8 h) HBF study (Unpublished Observations). Whether this association persists in space is unknown (Green & Scott, 2018) – although stature increments (Brown, 1977) and back pain (Kerstman et al., 2012; Pool‐Goudzwaard et al., 2015; Wing et al., 1991) are significant. However, no such correlations were observed for thoracic or cervical segments.

Increased stature in this study was positively correlated with anterior lumbar IVD height (L5–S1, L4–L5, L3–L4, L3–L2). It suggests the spine's elongation during spinal unloading leads to the expansion and thickening of these discs, contributing the overall height. Correlation results, while suggestive of relationships, cannot be solely relied upon for conclusive inference. Further experimental studies or longitudinal designs are needed to establish causal relationships.

Back pain also positively correlated with change in L2–L3, L3–L4 and L5–S1 anterior IVD heights. Whilst increments of IVD have been reported with DI (Treffel et al., 2017), to our knowledge this is the first time unloading‐induced back pain has been shown to correlate with IVD geometry changes.

Given moderate back pain induced by HBF, the low participant number and the inherent challenges of ultrasound and subjective ratings, the observation of positive correlations is surprising – suggestive that the back pain may be discogenic. Whether similar relationships exist in orbit is unknown, as the only study investigating IVD geometry reported no significant changes (Garcia et al., 2018). Passive VS failed to correlate with stature, back pain or any IVD measures. However, as there were no significant changes in passive VS this is unsurprising. That said, active VS is potentially a more sensitive parameter, and thus warrants further study.

### 4.6 Limitations and future studies

The fact that loading had similar effects suggests that the static squat was mild. Thirty seconds of 50% BW was defined as the axial ‘dose’ in order to represent that associated with use of ARED during an ISS resistive exercise training session (Petersen et al., 2016). However, ARED exercises generate high instantaneous forces. Therefore, future studies should attempt to better replicate ARED‐like loading.

Significant variability in some parameters suggests that larger sample sizes are warranted, particularly given the magnitude of the HBF effect is lower than that previously reported after 8 h. In our study body mass was included as a co‐variate. However, other additional covariates such as activity level and hydration status may also play a role and warrant exploration. In addition to active VS, muscle activation was not directly measured in our study but would be informative.

### 4.7 Conclusion

Four hours of HBF spinal unloading induced significant increments in standing stature, back pain and anterior IVD height at all levels from L2 to S1 (assessed by ISS‐compatible ultrasound) in all individuals across both groups supporting its validity as a novel analogue of acute spinal responses to microgravity. Stature, induced back pain and anterior IVD height increments were similarly (albeit not entirely) attenuated by 15 min upright seating and 30 s 50% BW axial loading. In contrast to previous simulated hypogravity studies, VS was unchanged by unloading or re‐loading. However, this may relate to the assessment of passive, rather than active (loaded) VS, and thus warrants further study. HBF‐induced back pain and stature positively correlated with lumbar IVD height indicating that IVD geometric changes may, at least in part, underpin space adaptation back pain and stature increments that can be rapidly modulated by brief periods of axial loading.

## AUTHOR CONTRIBUTIONS

D. Marcos‐Lorenzo, D. A. Green, J. Swanenburg, J. E. Clark, C. Lysandrou, and A. Gil‐Martinez conceived and designed the study; D. Marcos‐Lorenzo, C. Lysandrou, and L. Sudres performed the data collection; D. Marcos‐Lorenzo, D. A. Green, and J. Swanenburg analysed the data. D. Marcos‐Lorenzo and D. A. Green wrote the manuscript. All authors have read and approved the final version of this manuscript and agree to be accountable for all aspects of the work in ensuring that questions related to the accuracy or integrity of any part of the work are appropriately investigated and resolved. All persons designated as authors qualify for authorship, and all those who qualify for authorship are listed.

## CONFLICT OF INTEREST

All authors declare they have no competing interests.

## Data Availability

The datasets generated during and/or analysed during the current study are available at Figshare (https://doi.org/10.6084/m9.figshare.6025748.v1) and from the corresponding author on reasonable request.

## References

[eph13695-bib-0001] Andreoni, G. , Rigotti, C. , Baroni, G. , Ferrigno, G. , Colford, N. A. , & Pedotti, A. (2000). Quantitative analysis of neutral body posture in prolonged microgravity. Gait & Posture, 12(3), 235–242. 11154934 10.1016/s0966-6362(00)00088-6

[eph13695-bib-0002] Bailey, J. F. , Nyayapati, P. , Johnson, G. T. A. , Dziesinski, L. , Scheffler, A. W. , Crawford, R. , Scheuring, R. , O'Neill, C. W. , Chang, D. , Hargens, A. R. , & Lotz, J. C. (2022). Biomechanical changes in the lumbar spine following spaceflight and factors associated with postspaceflight disc herniation. The Spine Journal, 22(2), 197–206. 34343665 10.1016/j.spinee.2021.07.021

[eph13695-bib-0003] Belavý, D. L. , Armbrecht, G. , & Felsenberg, D. (2012). Incomplete recovery of lumbar intervertebral discs 2 years after 60‐day bed rest. Spine, 37(14), 1245–1251. 21971124 10.1097/BRS.0b013e3182354d84

[eph13695-bib-0003a] Belavý, D. L. , Armbrecht, G. , Richardson, C. A. , Felsenberg, D. , & Hides, J. A. (2011). Muscle atrophy and changes in spinal morphology: is the lumbar spine vulnerable after prolonged bed‐rest? Spine (Phila Pa 1976), 36(2), 137–145. 20595922 10.1097/BRS.0b013e3181cc93e8

[eph13695-bib-0004] Belavý, D. L. , Hides, J. A. , Wilson, S. J. , Stanton, W. , Dimeo, F. C. , Rittweger, J. , Felsenberg, D. , & Richardson, C. A. (2008). Resistive simulated weightbearing exercise with whole body vibration reduces lumbar spine deconditioning in bed‐rest. Spine, 33(5), E121–E131. 18317179 10.1097/BRS.0b013e3181657f98

[eph13695-bib-0005] Bobak, C. , Barr, P. , & O'Malley, A. (2018). Estimation of an inter‐rater intra‐class correlation coefficient that overcomes common assumption violations in the assessment of health measurement scales. BioMed Central Medical Research Methodology, 18(1), 93. 30208858 10.1186/s12874-018-0550-6 PMC6134634

[eph13695-bib-0006] Breen, A. , Carvil, P. , Green, D. A. , Russomano, T. , & Breen, A. (2023). Effects of a microgravity SkinSuit on lumbar geometry and kinematics. European Spine Journal, 32(3), 839–847. 36645514 10.1007/s00586-022-07454-x

[eph13695-bib-0007] Brown, J. (1977). ‘The Apollo‐Soyuz Test Project Medical Report National Aeronautics and Space Administration’. National Aeronautics and Space Administration.

[eph13695-bib-0008] Burkhart, K. , Allaire, B. , & Bouxsein, M. L. (2019). Negative effects of long‐duration spaceflight on paraspinal muscle morphology. Spine, 44(12), 879–886. 30624302 10.1097/BRS.0000000000002959

[eph13695-bib-0009] Carvil, P. A. , Attias, J. , Evetts, S. N. , Waldie, J. M. , & Green, D. A. (2017). The effect of the gravity loading countermeasure skinsuit upon movement and strength. Journal of Strength and Conditioning Research, 31(1), 154–161. 27135470 10.1519/JSC.0000000000001460

[eph13695-bib-0011] Carvil, P. A. , Russomano, T. , Halson‐Brown, S. , & Green, D. A. (2017). The effect of 4‐hour skinsuit induced partial axial reloading upon stature elongation and anterior intervertebral disc height as assessed by ultrasound after 8‐hour hyper‐buoyancy flotation. In 49th Annual Scientific Meeting of the British Medical Ultrasound Society .

[eph13695-bib-0013] Chang, D. G. , Healey, R. M. , Snyder, A. J. , Sayson, J. V. , Macias, B. R. , Coughlin, D. G. , Bailey, J. F. , Parazynski, S. E. , Lotz, J. C. , & Hargens, A. R. (2016). Lumbar spine paraspinal muscle and intervertebral disc height changes in astronauts after long‐duration spaceflight on the international space station. Spine, 41(24), 1917–1924. 27779600 10.1097/BRS.0000000000001873 PMC5588025

[eph13695-bib-0014] De Martino, E. , Hides, J. , Elliott, J. M. , Hoggarth, M. , Zange, J. , Lindsay, K. , Debuse, D. , Winnard, A. , Beard, D. , Cook, J. A. , Salomoni, S. E. , Weber, T. , Scott, J. , Hodges, P. W. , & Caplan, N. (2021). Lumbar muscle atrophy and increased relative intramuscular lipid concentration are not mitigated by daily artificial gravity after 60‐day head‐down tilt bed rest. Journal of Applied Physiology, 131(1), 356–368. 34080918 10.1152/japplphysiol.00990.2020

[eph13695-bib-0015] De Martino, E. , Salomoni, S. E. , Winnard, A. , McCarty, K. , Lindsay, K. , Riazati, S. , Weber, T. , Scott, J. , Green, D. A. , Hides, J. , Debuse, D. , Hodges, P. W. , van Dieën, J. H. , & Caplan, N. (2020). Hypogravity reduces trunk admittance and lumbar muscle activation in response to external perturbations. Journal of Applied Physiology, 128(4), 1044–1055. Erratum in: Journal of Applied Physiology. 2020 Jun 1;128(6):1684. PMID: 32163325; PMCID: PMC7191503. 32163325 10.1152/japplphysiol.00756.2019 PMC7191503

[eph13695-bib-0017] Garcia, K. M. , Harrison, M. F. , Sargsyan, A. E. , Ebert, D. , & Dulchavsky, S. A. (2018). Real‐time ultrasound assessment of astronaut spinal anatomy and disorders on the international space station. Journal of Ultrasound in Medicine, 37(4), 987–999. 28960477 10.1002/jum.14438

[eph13695-bib-0019] Girod, B. , Rabenstein, R. , & tenger, A. (1997). Einführung in Die Systemtheorie. Einführung in Die Systemtheorie. 10.1007/978-3-663-09883-6

[eph13695-bib-0020] Glaus, L. S. , Hofstetter, L. , Guekos, A. , Schweinhardt, P. , & Swanenburg, J. (2021). In vivo measurements of spinal stiffness according to a stepwise increase of axial load. European Journal of Applied Physiology, 121(8), 2277–2283. 33956197 10.1007/s00421-021-04705-5 PMC8260401

[eph13695-bib-0022] Green, D. A. , & Scott, J. P. R. (2018). Spinal Health during Unloading and Reloading Associated with Spaceflight. Frontiers in Physiology, 8, 1126. 29403389 10.3389/fphys.2017.01126 PMC5778142

[eph13695-bib-0022a] Hargens, A. R. , & Vico, L. (2016). Long‐duration bed rest as an analog to microgravity. Journal of Applied Physiology (1985), 120(8), 891–903. 10.1152/japplphysiol.00935.2015 26893033

[eph13695-bib-0024] Häusler, M. , Hofstetter, L. , Schweinhardt, P. , & Swanenburg, J. (2020). Influence of body position and axial load on spinal stiffness in healthy young adults. European Spine Journal, 29(3), 455–461. 31848714 10.1007/s00586-019-06254-0

[eph13695-bib-0025] Hides, J. A. , Lambrecht, G. , Sexton, C. T. , Pruett, C. , Petersen, N. , Jaekel, P. , Rosenberger, A. , & Weerts, G. (2021). The effects of exposure to microgravity and reconditioning of the lumbar multifidus and anterolateral abdominal muscles: Implications for people with LBP. The Spine Journal, 21(3), 477–491. 32966906 10.1016/j.spinee.2020.09.006

[eph13695-bib-0026] Hodges, P. W. , Cholewicki, J. , & van Dieen, J. H. (2013). Spinal control: The rehabilitation of back pain e‐book: state of the art and science. Elsevier Health Sciences.

[eph13695-bib-0027] Hofstetter, L. , Häusler, M. , Schweinhardt, P. , Heggli, U. , Bron, D. , & Swanenburg, J. (2021). Influence of axial load and a 45‐degree flexion head position on cervical spinal stiffness in healthy young adults. Frontiers in Physiology, 12, 786625. 35002768 10.3389/fphys.2021.786625 PMC8733818

[eph13695-bib-0028] Hofstetter, L. , Häusler, M. , Wirth, B. , & Swanenburg, J. (2018). Instrumented measurement of spinal stiffness: A systematic literature review of reliability. Journal of Manipulative and Physiological Therapeutics, 41(8), 704–711. 30612717 10.1016/j.jmpt.2018.03.002

[eph13695-bib-0030] Johnston, S. L. , Campbell, M. R. , Scheuring, R. , & Feiveson, A. H. (2010). Risk of herniated nucleus pulposus among U.S. astronauts. Aviation Space and Environmental Medicine, 81(6), 566–574. 20540448 10.3357/asem.2427.2010

[eph13695-bib-0031] Kerstman, E. L. , Scheuring, R. A. , Barnes, M. G. , DeKorse, T. B. , & Saile, L. G. (2012). Space adaptation back pain: A retrospective study. Aviation Space and Environmental Medicine, 83(1), 2–7. 22272509 10.3357/asem.2876.2012

[eph13695-bib-0032] Kingsley, M. I. , D'Silva, L. A. , Jennings, C. , Humphries, B. , Dalbo, V. J. , & Scanlan, A. T. (2012). Moderate‐intensity running causes intervertebral disc compression in young adults. Medicine and Science in Sports and Exercise, 44(11), 2199–2204. 22648342 10.1249/MSS.0b013e318260dbc1

[eph13695-bib-0033] Kozlovskaya, I. B. , Grigoriev, A. I. , & Stepantzov, V. I. (1995). Countermeasure of the negative effects of weightlessness on physical systems in long‐term space flights. Acta Astronautica, 36(8‐12), 661–668. 11541002 10.1016/0094-5765(95)00156-5

[eph13695-bib-0034] Leach, R. A. , Parker, P. L. , & Veal, P. S. (2003). PulStar differential compliance spinal instrument: A randomized interexaminer and intraexaminer reliability study. Journal of Manipulative and Physiological Therapeutics, 26(8), 493–501. 14569215 10.1016/S0161-4754(03)00106-4

[eph13695-bib-0035] Ledsome, J. R. , Lessoway, V. , Susak, L. E. , Gagnon, F. A. , Gagnon, R. , & Wing, P. C. (1996). Diurnal changes in lumbar intervertebral distance, measured using ultrasound. Spine, 21(14), 1671–1675. 8839471 10.1097/00007632-199607150-00012

[eph13695-bib-0038] Marcos‐Lorenzo, D. , Frett, T. , Gil‐Martinez, A. , Speer, M. , Swanenburg, J. , & Green, D. A. (2022). Effect of trunk exercise upon lumbar IVD height and vertebral compliance when performed supine with 1 g at the CoM compared to upright in 1 g. BioMed Central Sports Science, Medicine & Rehabilitation, 14(1), 177. 10.1186/s13102-022-00575-2 PMC9540696 36207739

[eph13695-bib-0039] Marshburn, T. H. , Hadfield, C. A. , Sargsyan, A. E. , Garcia, K. , Ebert, D. , & Dulchavsky, S. A. (2014). New heights in ultrasound: First report of spinal ultrasound from the international space station. Journal of Emergency Medicine, 46(1), 61–70. 24135505 10.1016/j.jemermed.2013.08.001

[eph13695-bib-0040] McKenzie, R. (2003). The lumbar spine: Mechanical diagnosis and therapy (p. 374).

[eph13695-bib-0041] McNamara, K. P. , Greene, K. A. , Moore, A. M. , Lenchik, L. , & Weaver, A. A. (2019). Lumbopelvic muscle changes following long‐duration spaceflight. Frontiers in Physiology, 10, 627. 31164840 10.3389/fphys.2019.00627 PMC6536568

[eph13695-bib-0042] Navasiolava, N. M. , Custaud, M. A. , Tomilovskaya, E. S. , Larina, I. M. , Mano, T. , Gauquelin‐Koch, G. , Gharib, C. , & Kozlovskaya, I. B. (2011). Long‐term dry immersion: Review and prospects. European Journal of Applied Physiology, 111(7), 1235–1260. 21161267 10.1007/s00421-010-1750-x

[eph13695-bib-0043] NCD Risk Factor Collaboration (NCD‐RisC) . (2016). A century of trends in adult human height. eLife, 5, e13410. 27458798 10.7554/eLife.13410 PMC4961475

[eph13695-bib-0044] Neuschwander, T. B. , Cutrone, J. , Macias, B. R. , Cutrone, S. , Murthy, G. , Chambers, H. , & Hargens, A. R. (2010). The effect of backpacks on the lumbar spine in children: A standing magnetic resonance imaging study. Spine, 35(1), 83–88. 20023607 10.1097/BRS.0b013e3181b21a5d

[eph13695-bib-0045] Nicogossian, A. E. , Williams, R. S. , Huntoon, C. L. , Doarn, C. R. , Polk, J. D. , & Schneider, V. S. (2016). Space Physiology and Medicine: From Evidence to Practice, Fourth Edition. Springer. 10.1007/978-1-4939-6652-3

[eph13695-bib-0048] Penchev, R. , Scheuring, R. A. , Soto, A. T. , Miletich, D. M. , Kerstman, E. , & Cohen, S. P. (2021). Back Pain in Outer Space. Anesthesiology, 135(3), 384–395. 33979426 10.1097/ALN.0000000000003812

[eph13695-bib-0049] Petersen, N. , Jaekel, P. , Rosenberger, A. , Weber, T. , Scott, J. , Castrucci, F. , Lambrecht, G. , Ploutz‐Snyder, L. , Damann, V. , Kozlovskaya, I. , & Mester, J. (2016). Exercise in space: The European Space Agency approach to in‐flight exercise countermeasures for long‐duration missions on ISS. Extreme Physiology & Medicine, 5, 9. 27489615 10.1186/s13728-016-0050-4 PMC4971634

[eph13695-bib-0050] Plehuna, A. , Green, D. A. , Amirova, L. E. , Tomilovskaya, E. S. , Rukavishnikov, I. V. , & Kozlovskaya, I. B. (2022). Dry immersion induced acute low back pain and its relationship with trunk myofascial viscoelastic changes. Frontiers in Physiology, 13, 1039924. 36311233 10.3389/fphys.2022.1039924 PMC9606241

[eph13695-bib-0051] Pool‐Goudzwaard, A. L. , Belavý, D. L. , Hides, J. A. , Richardson, C. A. , & Snijders, C. J. (2015). Low back pain in microgravity and bed rest studies. Aerospace Medicine and Human Performance, 86(6), 541–547. 26099126 10.3357/AMHP.4169.2015

[eph13695-bib-0052] Qin, B. , Baldoni, M. , Wu, B. , Zhou, L. , Qian, Z. , & Zhu, Q. (2022). Effect of lumbar muscle atrophy on the mechanical loading change on lumbar intervertebral discs. Journal of Biomechanics, 139, 111120. 35588559 10.1016/j.jbiomech.2022.111120

[eph13695-bib-0053] Rathinam, C. , Bridges, S. , Spokes, G. , & Green, D. A. (2013). ‘Effects of Lycra body suit orthosis on a child with developmental coordination disorder: A case study’. Journal of Prosthetics and Orthotics, 25(1), 58–61.

[eph13695-bib-0055] Sayson, J. V. , & Hargens, A. R. (2008). Pathophysiology of low back pain during exposure to microgravity. Aviation Space and Environmental Medicine, 79(4), 365–373. 18457293 10.3357/asem.1994.2008

[eph13695-bib-0056] Sayson, J. V. , Lotz, J. C. , Parazynski, S. E. , Change, D. G. , Healey, R. M. , & argens, A. R. (2015). 'Microgravity‐Induced Back Pain and Intervertebral Disc Herniation: International Space Station Results.

[eph13695-bib-0058] Scott, J. P. R. , Weber, T. , & Green, D. A. (2019). Introduction to the frontiers research topic: Optimization of exercise countermeasures for human space flight – Lessons from terrestrial physiology and operational considerations. Frontiers in Physiology, 10, 173. 30899226 10.3389/fphys.2019.00173 PMC6416179

[eph13695-bib-0059] Shymon, S. , Hargens, A. R. , Minkoff, L. A. , & Chang, D. G. (2014). Body posture and backpack loading: An upright magnetic resonance imaging study of the adult lumbar spine. European Spine Journal, 23(7), 1407–1413. 24619606 10.1007/s00586-014-3247-5 PMC6339990

[eph13695-bib-0060] Sobczak, S. , Dugailly, P. M. , Gilbert, K. K. , Hooper, T. L. , Sizer, P. S., Jr , James, C. R. , Poortmans, B. , Matthijs, O. C. , & Brismée, J. M. (2016). Reliability and validation of in vitro lumbar spine height measurements using musculoskeletal ultrasound: A preliminary investigation. Journal of Back and Musculoskeletal Rehabilitation, 29(1), 171–182. 26406194 10.3233/BMR-150613

[eph13695-bib-0060a] Stabler, R. A. , Rosado, H. , Doyle, R. , Negus, D. , Carvil, P. A. , Kristjánsson, J. G. , Green, D. A. , Franco‐Cendejas, R. , Davies, C. , Mogensen, A. , Scott, J. , & Taylor, P. W. (2017). Impact of the Mk VI SkinSuit on skin microbiota of terrestrial volunteers and an International Space Station‐bound astronaut. Nature Partner Journals Microgravity, 3, 23. 10.1038/s41526-017-0029-5 PMC5589758 28894789

[eph13695-bib-0061] Stokes, I. A. , & Gardner‐Morse, M. (2003). Spinal stiffness increases with axial load: Another stabilizing consequence of muscle action. Journal of Electromyography and Kinesiology, 13(4), 397–402. 12832169 10.1016/s1050-6411(03)00046-4

[eph13695-bib-0062] Styf, J. R. , Ballard, R. E. , Fechner, K. , Watenpaugh, D. E. , Kahan, N. J. , & Hargens, A. R. (1997). Height increase, neuromuscular function, and back pain during 6 degrees head‐down tilt with traction. Aviation Space and Environmental Medicine, 68(1), 24–29. 9006878

[eph13695-bib-0063] Sundblad, P. , Orlov, O. , Angerer, O. , Larina, I. , & Cromwell, R. (2014). Guidelines for standardization of bed rest studies in the spaceflight context. www.iaaweb.org 10.1152/japplphysiol.00089.2016 26917693

[eph13695-bib-0064] Swanenburg, J. , Easthope, C. A. , Meinke, A. , Langenfeld, A. , Green, D. A. , & Schweinhardt, P. (2023). Lunar and mars gravity induce similar changes in spinal motor control as microgravity. Frontiers in Physiology, 14, 1196929. 37565140 10.3389/fphys.2023.1196929 PMC10411353

[eph13695-bib-0065] Swanenburg, J. , Langenfeld, A. , Easthope, C. A. , Meier, M. L. , Ullrich, O. , & Schweinhardt, P. (2020). Microgravity and hypergravity induced by parabolic flight differently affect lumbar spinal stiffness. Frontiers in Physiology, 11, 562557. 32982803 10.3389/fphys.2020.562557 PMC7492749

[eph13695-bib-0066] Thornton, W. , Hoffler, G. W. , & Rummel, J. (1977). ‘Anthropometric changes and fluid shifts’.

[eph13695-bib-0067] Treffel, L. , Massabuau, N. , Zuj, K. , Custaud, M. A. , Gauquelin‐Koch, G. , Blanc, S. , Gharib, C. , & Millet, C. (2017). Pain and vertebral dysfunction in dry immersion: A model of microgravity simulation different from bed rest studies. Pain Research & Management, 2017, 9602131. 28785161 10.1155/2017/9602131 PMC5530446

[eph13695-bib-0068] Tyrrell, A. R. , Reilly, T. , & Troup, J. D. (1985). Circadian variation in stature and the effects of spinal loading. Spine, 10(2), 161–164. 4002039 10.1097/00007632-198503000-00011

[eph13695-bib-0069] Waldie, J. M. , & Newman, D. J. (2011). ‘A gravity loading countermeasure skinsuit’. Acta Astronautica, 68(7–8), 722–730.

[eph13695-bib-0070] Wing, P. C. , Tsang, I. K. , Susak, L. , Gagnon, F. , Gagnon, R. , & Potts, J. E. (1991). Back pain and spinal changes in microgravity. Orthopedic Clinics of North America, 22(2), 255–262. 1826549

[eph13695-bib-0071] Wotring, V. E. (2015). Medication use by U.S. crewmembers on the International Space Station. Federation of American Societies for Experimental Biology Journal, 29(11), 4417–4423. 26187345 10.1096/fj.14-264838

[eph13695-bib-0072] Young, K. S. , Kim, K. H. , & Rajulu, S. (2021). Anthropometric changes in spaceflight. Human Factors, 22, 187208211049008. 10.1177/00187208211049008 34674563

